# Conservative Resolution of a Vesicovaginal Fistula Including Laser Therapy in a Patient Who Underwent Recurrent Surgery After Prior Radiotherapy for Endometrial Cancer

**DOI:** 10.1002/cnr2.70056

**Published:** 2024-11-19

**Authors:** Alessandro Buda, Jessica Mauro, Francesco Varvello, Jacopo Antolini, Giuseppe Di Guardia, Enrica Bar, Federica Filipello, Rodolfo Milani

**Affiliations:** ^1^ Division of Gynecologic Oncology Michele and Pietro Ferrero Hospital Verduno Italy; ^2^ Division of Urology Michele and Pietro Ferrero Hospital Verduno Italy; ^3^ Division of Radiology Michele and Pietro Ferrero Hospital Verduno Italy; ^4^ Division of Pathology Michele and Pietro Ferrero Hospital Verduno Italy; ^5^ Consultant Professor for the Division of Gynecologic Oncology Michele and Pietro Ferrero Hospital Verduno Italy

**Keywords:** endometrial cancer, laser therapy, previous‐irradiation, recurrence, robotic partial colpectomy, vesicovaginal fistula

## Abstract

**Background:**

Isolated vaginal vault recurrence of endometrial cancer can be treated with rescue radiotherapy. However, in previously irradiated patients, surgical resection can be considered the treatment of choice. Vesicovaginal fistulas (VVFs) sometimes complicate the surgical intervention because of the presence of massive ischemia and fibrosis of pelvic tissue from previous irradiation. Traditional strategies for the treatment of VVFs include endoscopic treatment (when feasible) or a laparoscopic, robotic, or open abdominal approach in some experiences through a transvesical route. The last approach can be associated with long inpatient hospital stays, postoperative complications, and failure, especially in obese patients. Our report proposes a conservative approach with prolonged catheterization and placement of nephrostomy tubes to treat a VVF with laser therapy of the fistula.

**Case:**

We present the case of a woman with a second relapse of endometrial cancer at the level of the vaginal vault, after a hysterectomy and then radiotherapy for a first relapse, who underwent robotic partial colpectomy, with an intraoperative bladder lesion, which was repaired with interrupted stitches. However, the patient developed a vesicovaginal fistula. A conservative approach was initially undertaken as an alternative to the surgical repair of the fistula. After the clinical and radiological confirmation of the fistula andconsidering the patient's clinical condition, the multidisciplinary team proposed a conservative management of the fistula as an alternative to fistula surgical repair. Bladder catheter Ch 20 and bilateral nephrostomy did not completely resolve the fistula, with a minor residual linkage between the bladder and the vaginal vault after 8 months from the robotic surgery. A single/month diode laser application for 3 months was added to the conservative treatment. Cystography was negative at the end of laser sessions, and both nephrostomies were removed 1 week later. After 6 months, clinical and radiological follow‐up was negative, and no further vaginal urine loss was recorded.

**Conclusion:**

We believe that conservative management of a complex vesicovaginal fistula after multiple treatments for endometrial cancer is possible. In this scenario, laser therapy can be a valuable clinical tool to improve the outcome, with reduced invasiveness for the patient.

## 1 Introduction

Endometrial cancer with an estimated 417 000 new cases in 2020 represents the sixth most common cancer occurring in women [1]. Women with apparent‐confined uterine disease are surgically treated with extrafascial hysterectomy, bilateral salpingo‐oophorectomy, and sentinel lymph node biopsy [2]. Current guidelines establish that for patients with low‐risk endometrial cancer, no adjuvant treatment is recommended [3]. However, 7% to 15% of patients with early‐stage disease can experience a recurrence [4, 5]. The rate of isolated vaginal recurrences is low, and in the case of non‐irradiated patients, salvaged treatment includes brachytherapy [6, 7]. However, surgical resection can be considered in previously irradiated patients as the first‐line treatment option [8, 9]. Vesicovaginal fistulas (VVFs) are relatively rare in the developed world, primarily occurring after simple or radical hysterectomy and radiotherapy [10, 11]. Usually, the surgical repair rate of fistulas occurring as a surgical complication is high, ranging between 75% and 95%, [12] Despite this, when VVFs occur in previously irradiated patients, their management is a huge challenge. Fistula repair in the radiotherapy field potentially involves tissues widely affected by ischemia, fibrosis, and contracture [13]. The presentation and outcomes of VVFs occurring within the radiotherapy‐treated pelvis are scanty in the literature. Treatment options require a multidisciplinary approach and should be tailored to patients' individual conditions [14, 15]. Vaginal laser therapy has been used in the treatment of genitourinary syndrome of menopause or vaginal atrophy. It has been shown to promote the activation of fibroblasts in the vaginal matrix and encourage the deposition of collagen and elastic fibers, which leads to its angiogenesis and tissue reshaping [16]. Furthermore, laser therapy has been evaluated for the treatment of rectovaginal fistulas with promising results [17].

However, there are no available reports describing the conservative resolution of a VVF with the use of vaginal laser therapy in addition to conservative management including urine diversion. We present the conservative management of a case of VVF after robotic partial colpectomy in a patient presenting a vaginal vault recurrence of previously irradiated low‐risk endometrial cancer.

## 2 Case Presentation

A 68‐year‐old woman with a history of endometrial cancer experienced a vaginal vault recurrence. Chronic stable medical problems included hypertension, insulin‐dependent diabetes mellitus, and class II obesity (body mass index of 39.6 kg/m^2^).

She was diagnosed with endometrial hyperplasia with atypia in 2015 and therefore underwent a total hysterectomy and bilateral salpingo‐oophorectomy by an open approach. The final pathology report diagnosed a low‐risk grade 1 endometrioid endometrial cancer FIGO stage IA. The follow‐up was uneventful for 6 years. In March 2021, during a clinical follow‐up evaluation in our hospital, the patient presented abnormal vaginal bleeding. During clinical examination, a 3 cm nodule was found in the vaginal vault, and the biopsy revealed an endometrial cancer recurrence.

**FIGURE 1:**
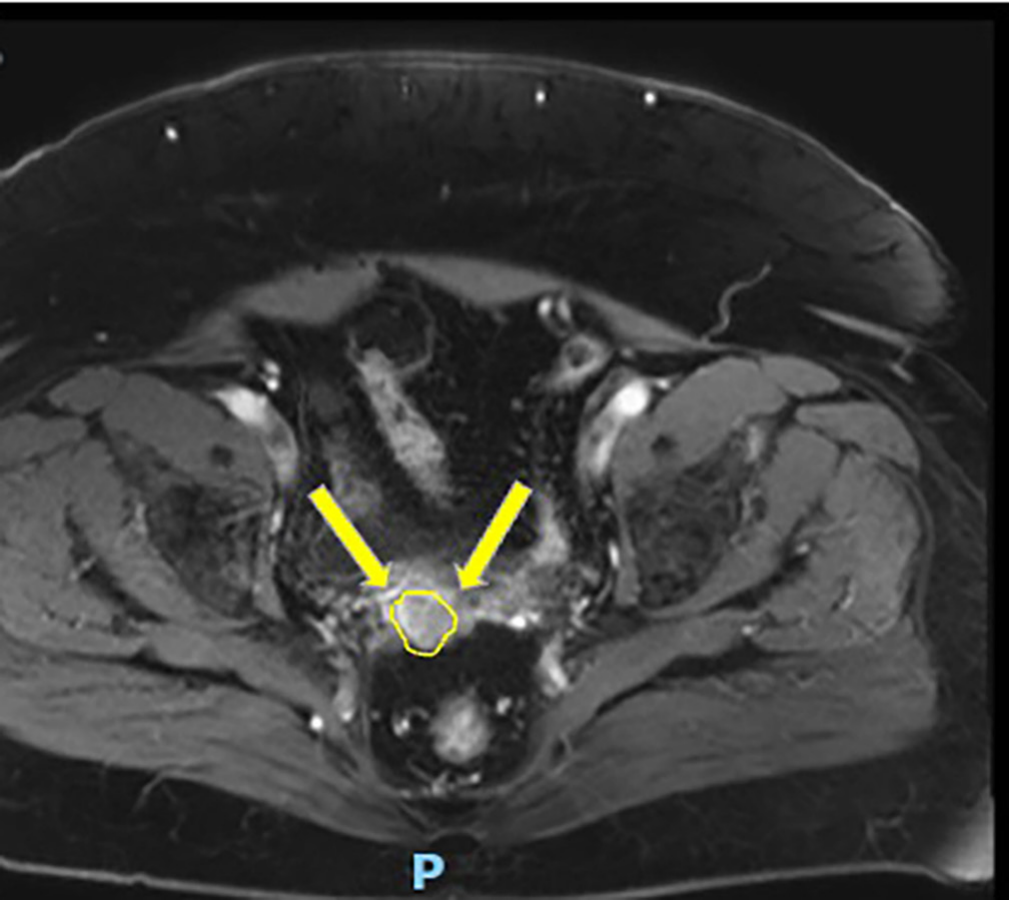
MRI image showing the first recurrence in the vaginal vault. Arrows highlight a nodule of 33×21×21 mm.

The CT scan was negative for distant or peritoneal metastasis, while the MRI showed the presence of a nodule of 33×21×21 mm in the vaginal vault (Figure 1).

The patients underwent exclusive pelvic external beam radiotherapy with a total dose of 65 Gy, with no residual disease at the end of the treatment.

A second vaginal vault recurrence with a palpable nodule of less than 1 cm was diagnosed in February 2023. The CT scan did not reveal any distant recurrence, and considering the previously irradiation field, in March 2023, she underwent a robotic‐assisted colpectomy of the vaginal vault including the upper third of the vagina.

A vaginal rigid insert was used to identify the cleavage planes between the vagina and the bladder and between the vagina and the rectum. At the end of the dissection of the bladder from the vagina, made complex by radiation‐induced fibrosis, a continuous vaginal cuff solution of about 1 cm was determined. The lesion was sutured in a single layer with a detached stitch suture of Vicryl 3/0. The vagina was sutured with Vicryl 2/0 with a continuous suture. The bladder‐filling test with methylene blue at the end of the surgery did not reveal any dye leakage. A 22 Ch silicone catheter was left in place. The whole surgical duration was 2.5 h, and the estimated blood loss was about 150 mL.

**FIGURE 2:**
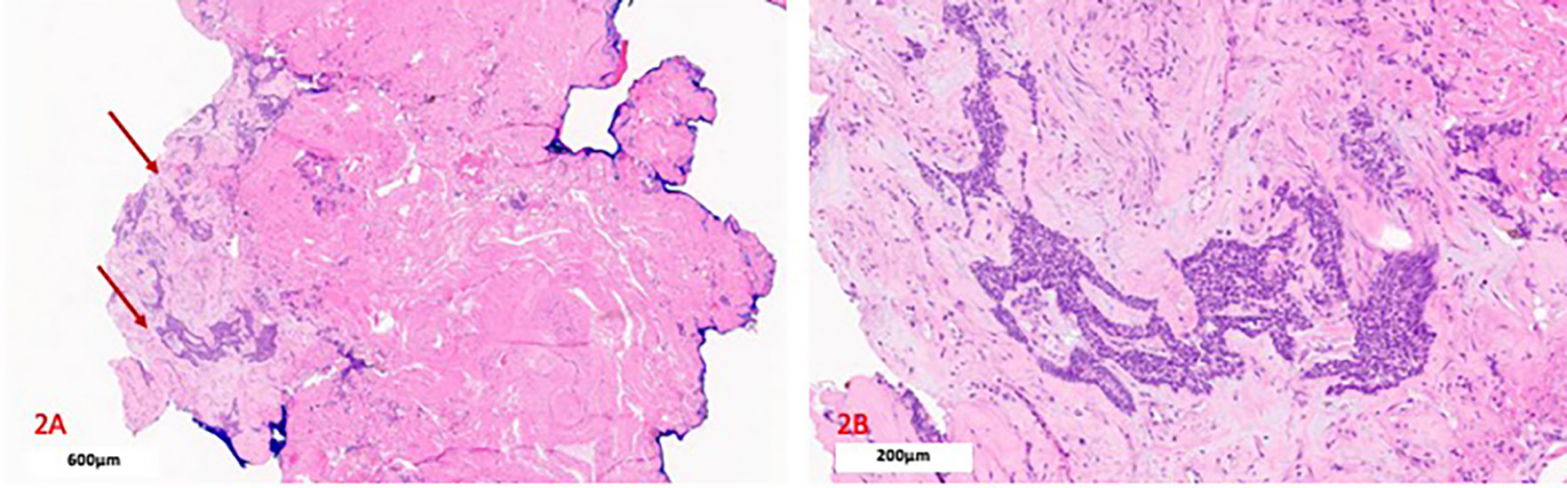
Final pathological examination confirming the second recurrence in the upper third of the vagina. (A) Low‐power view of the vaginal vault made of fibrosclerotic tissues with the glands of endometrial carcinoma (arrows). (*Hematoxylin and eosin‐ 600 μm H&E, 40X*). (B) Details of image A showing a higher‐power view of endometrial carcinoma. Neoplastic cells became immunoreactive for cytokeratin (CKAE1/AE3), estrogen, and progesterone (*Hematoxylin and eosin 200 μm H&E, 100X*).

The postoperative course was uneventful. The final pathology confirmeda low‐grade endometrioid recurrence in the vaginal vault with free margins (Figure 2).

**FIGURE 3:**
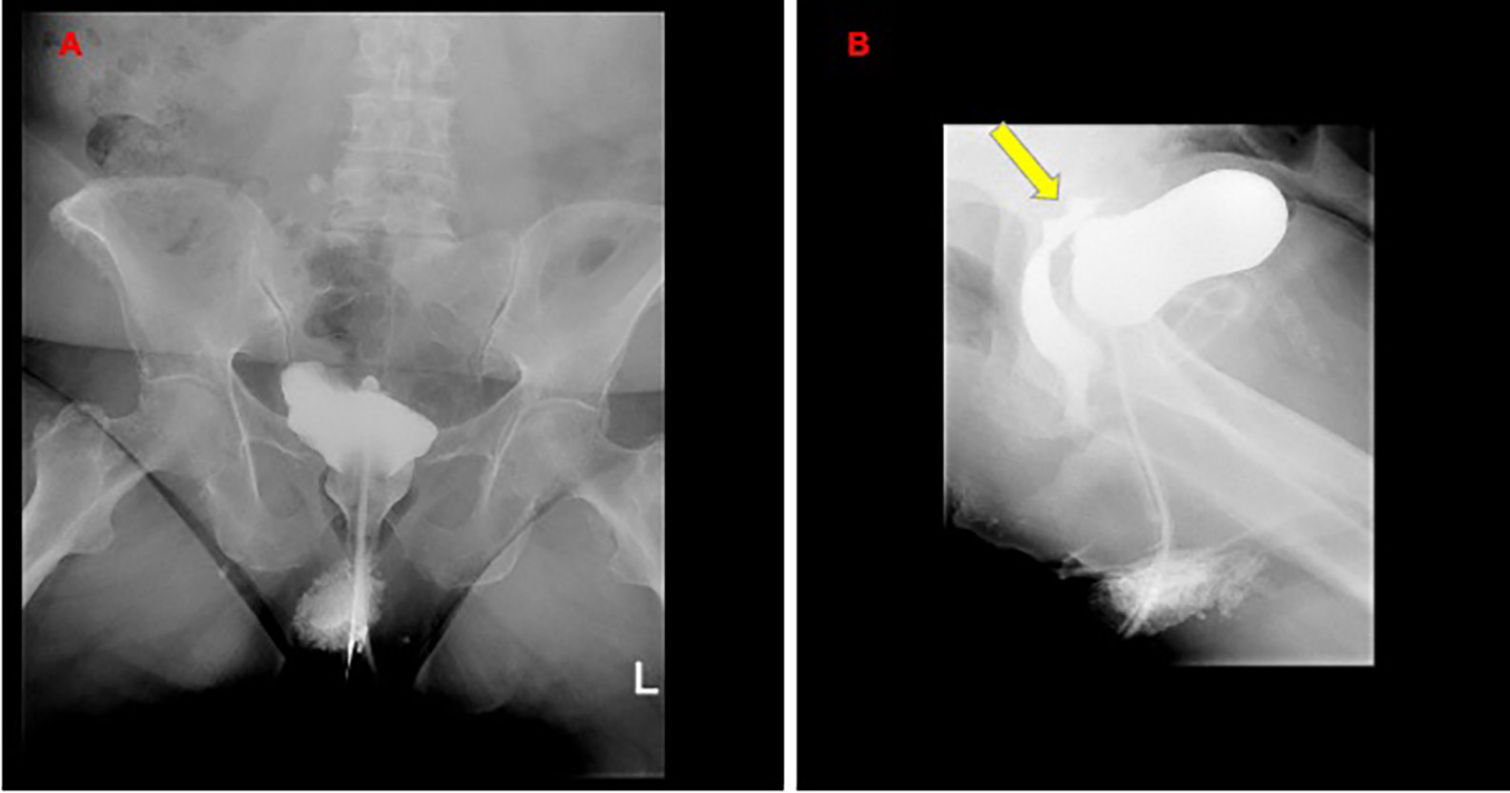
(A, B). Cystography RX images showing the presence of a large vesicovaginal fistula with a diameter of 11 mm in the lateral projection with 80 mL of the contrast agent present in the superior dorsal portion of the bladder (see the arrow in Figure 4B).

The patient was discharged on day 6 with a long‐term bladder catheter. Forty days after the surgery, the patient underwent a cystoscopy, which showed normal healing, and the bladder catheter was removed. One week later, the woman was readmitted to the hospital for urine vaginal discharge, and a vesicovaginal fistula was diagnosed. Cystography showed a VVF with a diameter of approximately 11 mm (Figure 3). Considering the patient's clinical condition, after a multidisciplinary discussion, conservative management of the fistula was proposed as an alternative to fistula repair surgery. Three weeks later, the cystoscopy examination confirmed the presence of a 10 mm fistula in the bladder base, and a bladder catheter 20 Ch was inserted with a bilateral mono J catheter.

In June 2023, in the presence of persistent vaginal urinary loss and an unsolved vesicovaginal fistula by diagnostic cystography, a bilateral nephrostomy was planned by the multidisciplinary team instead of a mono J catheter. However, nephrostomy placement was deferred because of the occurrence of pulmonary thromboembolism arising from a deep venous thrombosis of the femoral right vein. The therapeutic thrombolysis was promptly started.

Since the VVF did not spontaneously repair, an inferior vena cava filter was placed in September 2023, 2 months after the diagnosis of the pulmonary embolism.

**FIGURE 4:**
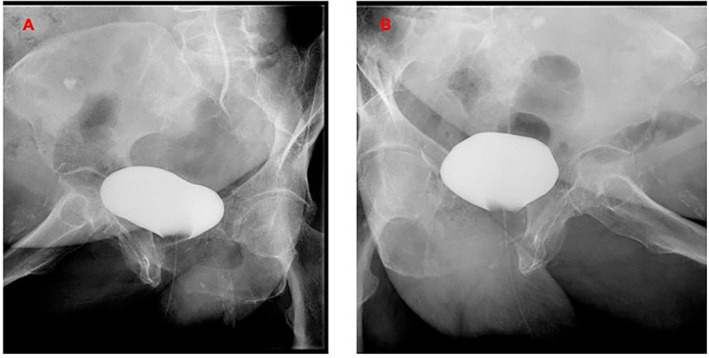
(A, B). Cystography RX images at the end of the conservative treatment in December 2023, showing no further bladder leakage into the vagina (bladder filled with 100 mL of the contrast agent).

During the subsequent clinical re‐evaluation, considering the improvement in the absence of urine leakage, the bladder catheter was removed in the absence of evident urine leakage from the fistula. The conservative protocol included local vaginal therapy of vitamin C and hyaluronic acid in addition to a diode laser/month application for 3 months. Vitamin C vaginal tablets were administered for 15 days per month, alternating with hyaluronic acid gels of 5 g per application on the remaining 15 days of the month. Three months later, cystography was negative at the end of the laser sessions (Figure 4), and both nephrostomies were removed 1 week later. After 6 months, clinical and radiological follow‐up was negative, and no further vaginal urine loss was recorded. The oncological follow‐up in September 2024 was negative in the absence of any evidence of recurrence.

## 3 Discussion

When a vaginal vault recurrence occurs in a previously irradiated patient, surgical resection represents the treatment of choice [18]. Surgical intervention is a challenge in the presence of tissue ischemia including the bladder base and pelvic fibrosis as well as the bowel. The vaginal approach can be an option to try to avoid a hostile pelvis and post‐irradiation fibrosis; however, the surgical approach should be tailored to each patient. In our case report, we choose a robotic‐assisted minimally invasive approach in the presence of severe obesity and co‐morbidity to try to reduce the post‐operative complications. Moreover, in this case, the vaginal approach was more complex because of the patient's habitus, and therefore, recurrence in the vaginal vault was not easily accessible.

Minimally invasive/robotic surgery to repair VVFs seems to be associated with less prolonged hospitalization, a lower rate of complications, and a lower rate of readmission when compared with the open approach [19]. However, in our opinion in this case the presence of a massive fibrosis of the pelvic tissues included the presence of the vaginal cuff and the tenacious adhesions present between rectus and vagina was determinant for the intraoperative bladder lesion that hesitate in a post‐operative VVF.

VVF closure in previously irradiated patients is a challenge, with an initial success rate of 20% and an overall success rate of only 25%. Fistulas that arise spontaneously are less likely to be repaired, and when repair is attempted, a success rate of about 40% is observed compared with 100% of post‐surgical fistulas. In addition, 70% required primary or secondary urinary diversion due to associated bladder dysfunction and ureteral strictures [14].

This was probably related to the poor viability of the irradiated bladder and was further complicated by a deep venous thrombosis with pulmonary embolism that delayed the treatment of the VVF including the bilateral nephrostomy.

The laser adjuvant treatment to support and enhance the VVF closure was proposed after the evidence of a minimal residual linkage between the bladder and the vaginal vault. The treatment was performed using a Leonardo dual diode laser (Biolitec Italia Srl, Milano, Italy) that features a combination of two wavelengths (980 nm and 1470 nm), which offers a multitude of interactions with tissues. At each session, the power was set to 7 watts in a pulsed mode (two pulses of 0.5 s each, with 0.5 s of pause). To reduce the local discomfort and pain, 2% lidocaine vaginal gel was administered, and after about 5 min, the dedicated laser fiber was inserted into a glass handpiece placed into the vagina. Eight pulses were applied at 2, 10, and 12 o'clock for every cm of the vaginal wall from the fornix to the introitus. The laser treatment was well tolerated for all the sessions without pain.

There is no definitive consensus in the scientific community on how to resolve small primary or residual fistulas. Some authors proposed a laser therapy for the treatment of VVFs less than 3 mm in the following conservative primary treatment or recured after primary surgical repair [20, 21].

In gynecology, laser technologies have been recently proposed as an alternative option for the treatment of genitourinary syndrome of menopause (GSM) and vulvovaginal atrophy (VVA). The thermal effect generated by non‐ablative diode lasers enhances vascularization and collagen production, avoiding most of the adverse effects on the vaginal epithelium by causing direct thermal damage only to the connective tissue, sparing the superficial epithelium [22, 23]. Recent data show that three sessions of a diode vaginal laser seem to be an effective and easily tolerated procedure for vaginal functional restoration in the treatment of GSM and VVA [24]. Based on these results, we decide to recommend the patients a three‐diode laser vaginal session that enhances and favors the complete closure of residual VVFs.

## 4 Conclusion

Management of urinary fistulas is a complex and challenging surgical procedure, requiring high surgical skills, detailed knowledge of the female pelvic anatomy, experience, and a flexible mindset of the operating surgeon. VVFs in irradiated patients are a huge challenge both for gynecologists and urologists. Conservative management of complex cases, like the one presented in this report, with the integrative approach of laser treatment, can offer a strategy for successful outcomes, even when the patient's characteristics and the severity of the local anatomical field pose unique challenges to the managing team.

## Author Contributions

**Alessandro Buda:** conceptualization, methodology, validation, funding acquisition, writing – original draft, writing – review and editing, project administration, supervision, formal analysis, resources. **Jessica Mauro:** conceptualization, investigation, writing – original draft, data curation, formal analysis, resources. **Francesco Varvello:** data curation, writing – review and editing. **Jacopo Antolini:** writing – review and editing, data curation. **Giuseppe Di Guardia:** visualization, data curation, software. **Enrica Bar:** data curation, visualization. **Federica Filipello:** visualization, data curation, writing – review and editing, software. **Rodolfo Milani:** conceptualization, writing – review and editing, supervision, data curation.

## Ethics Statement

The patient provided a written informed consent for the publication of this case report and accompanying images.

## Conflicts of Interest

The authors declare no conflicts of interest.

## Data Availability

Data sharing not applicable to this article as no datasets were generated or analysed during the current study.
